# Blastomycosis in Man after Kinkajou Bite

**DOI:** 10.3201/eid1702.101046

**Published:** 2011-02

**Authors:** Julie R. Harris, David D. Blaney, Mark D. Lindsley, Sherif R. Zaki, Christopher D. Paddock, Clifton P. Drew, April J. Johnson, Douglas Landau, Joel Vanderbush, Robert Baker

**Affiliations:** Author affiliations: Centers for Disease Control and Prevention, Atlanta, Georgia, USA (J.R. Harris, D.D. Blaney, M.D. Lindsley, S.A. Zaki, C.D. Paddock, C.P. Drew);; Purdue University School of Veterinary Medicine, West Lafayette, Indiana, USA (A.J. Johnson);; Indiana State Department of Health Laboratories, Indianapolis, Indiana, USA (D. Landau);; Animalia, Inc., Indianapolis (J. Vanderbush);; Community Health Network, Indianapolis (R. Baker)

**Keywords:** Blastomycosis, zoonoses, wound infection, fungi, kinkajou, human infection, dispatch

## Abstract

We report transmission of *Blastomyces dermatitidis* fungal infection from a pet kinkajou to a man. When treating a patient with a recalcitrant infection and a history of an animal bite, early and complete animal necropsy and consideration of nonbacterial etiologies are needed.

Blastomycosis is caused by inhalation of conidia of the dimorphic fungus *Blastomyces dermatitidis*. This fungus causes pneumonia; disseminated infection; or rarely, cutaneous disease through contact with a wound (*1*). It is endemic to southern, south-central, and midwestern states in the United States, particularly in areas bordering the Mississippi and Ohio Rivers (*2*). Outbreaks among humans have been linked to recreational activities near rivers or streams in disease-endemic areas (*3**,**4*).

Blastomycosis can also affect other mammals (*5*). Zoonotic transmission of blastomycosis is rare but has been reported in association with dog bites (*6**,**7*), cat scratches (*8*), and animal necropsies (*9*). We report zoonotic transmission of blastomycosis by a bite from a pet kinkajou.

## The Study

On September 21, 2009, a 37-year-old male zoologist in Indianapolis, Indiana, visited his physician with a 3-day history of swelling and tenderness of the third digit of his right hand. He reported having been bitten on the affected finger on August 29 by his pet kinkajou. At the time, the animal was severely ill with respiratory signs and died shortly after biting the patient. The wound initially healed after treatment with antimicrobial ointment. At the physician visit, the patient was prescribed 2 weeks of doxycycline and amoxicillin/clavulanate and instructed to return if no improvement was noted.

On September 24, the patient returned with worsening pain. He was hospitalized the next day with fever (101.0°F), nausea, headache, and continued finger tenderness. Ascending lymphangitis and swollen, tender, axillary lymph nodes were noted. Except for his leukocyte count (15,100 cells/mL), laboratory values were within reference ranges. The patient was treated with intravenous vancomycin and ampicillin/sulbactam, wound incision, and drainage. Results of blood and wound cultures were negative. By September 27, the patient’s fever and lymphangitis subsided. He was discharged and received amoxicillin/clavulanate and ciprofloxacin.

The next week the patient returned to the emergency department with erythematous nodules along his right basilic vein and recurrent ascending lymphadenitis. An infectious disease consultant noted fusiform swelling of the right middle finger, nodular erythematous fluctuant areas of the right wrist, swollen and tender axilla, and a nodular area on the right ankle. A punch biopsy sample from the right hand showed acute inflammation and suppurative granulomas of deep soft tissue. Initial stainings were negative for parasites, fungi, and acid-fast bacilli. Tissue and blood cultures for aerobic, anaerobic, acid-fast, and fungal organisms were negative, as were serologic results for *Bartonella* spp. and *Brucella* spp.

The patient was readmitted to a local hospital on October 12 with lesions on the right ankle and swelling of the left ankle. Results of complete blood count with differential, metabolic profile, and liver function studies were unremarkable; additional blood cultures were obtained. The patient was treated with azithromycin, ciprofloxacin, and streptomycin. Axilla aspirate was cultured. On October 16, the patient’s condition improved, and he was discharged. Blood cultures obtained during hospitalization were negative. However, a mold was found growing in the axillary fungal culture.

Tissue from the punch biopsy sample and mold cultures were sent to the Indiana State Department of Public Health Laboratories, where *B*. *dermatitidis* was isolated from culture on October 21. The patient was prescribed itraconazole (200 mg 2×/d) for 6 months; he improved rapidly, and his infection resolved completely.

Before this illness, the patient had been healthy and had no recent history of travel or recent history of camping, digging, or gardening. None of the >60 other exotic animals he cared for had a history of *B*. *dermatitidis* infection.

Kinkajous (*Potos flavus*), which are native to South and Central America, are members of the family Procyonidae. *Leishmania* spp (*10*), herpesvirus (*11*), and *Salmonella* spp (*12*). have been reported in association with kinkajous, but only *Kingella potus* has been reported as a zoonotically transmitted infection resulting from a kinkajou bite (*13*).

The kinkajou in this report was a 9-year-old wild-born female (birth location unknown) brought to the United States at ≈2 years of age by an animal facility in Texas. It was acquired by the patient in November 2008 from an educational organization in Chicago.

The kinkajou lived in a large walk-in enclosure in the patient’s basement with its male cage mate, which never displayed similar illness and remains alive. The enclosure included plastic platforms and a nest box with regularly cleaned T-shirt bedding, hanging hammock-style fleece sleep bags, and wooden branches collected from outside that were treated regularly with a bleach scrub. The kinkajou’s diet included fresh fruits, vegetables, and monkey crunch biscuits (Mazuri, Lincoln, NE, USA). It was not handled outside the home, did not roam outside its enclosure, and had no contact with other animals besides its cage mate. Its medical history was unremarkable. The patient reported that the kinkajou showed increased respiratory distress during the 3 days before its death.

The initial necropsy, performed in early September 2009, showed white lesions on the kinkajou’s lungs, which suggested bacterial pneumonia. However, no bacterium was cultured. No histopathologic examinations for rabies were performed at that time. Immediately after necropsy, the carcass was frozen. After the patient’s diagnosis of blastomycosis, the carcass was thawed, and lung and oral mucosal tissue samples were sent to the Centers for Disease Control and Prevention, Atlanta, GA, USA, for culture and molecular, histopathologic, and immunohistochemical (IHC) analysis.

**Figure 1:**
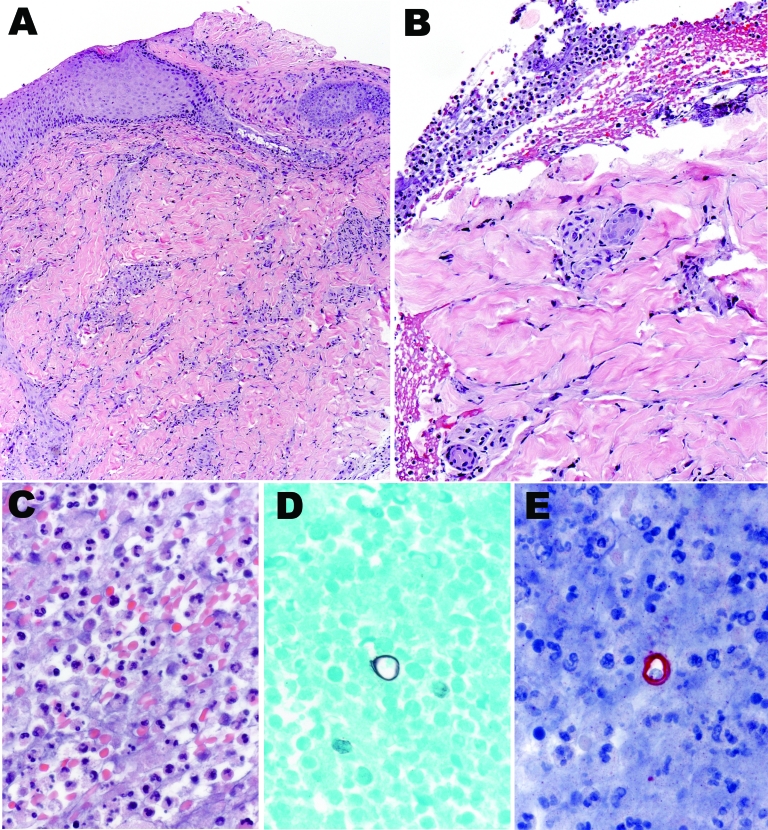
Histologic appearance of the cutaneous lesion of a man with blastomycosis. Ulcerated epidermis (A) showing superficial and deep perivascular infiltrates, predominantly mononuclear inflammatory cells. Fibrinopurulent exudate (B) adjacent to the ulcer, comprising neutrophils, erythrocytes, and necrotic cellular debris (C), and occasional large yeasts morphologically compatible with *Blastomyces dermatitidis* infection (D and E). Hematoxylin and eosin stain (A, B, and C), Grocott methenamine silver stain (D), and immunoalkaline phosphatase with antibody against *B*. *dermatitidis* and naphthol fast red with hematoxylin counterstain (E). Original magnifications ×12.5 (A), ×25 (B), and ×100 (C–E).

The Centers for Disease Control and Prevention received tissue samples from the kinkajou and patient punch biopsy samples. Sections of skin from the patient showed extensive epidermal ulceration with superficial and deep perivascular inflammatory cell infiltrates comprising predominantly lymphocytes and macrophages (Figure 1). A fibrinopurulent exudate containing neutrophils, erythrocytes, and necrotic cellular debris tracked from the deep dermis to the edge of the ulcer. Large, ovoid yeast cells with double-contoured walls, ≈10–15 µm, were identified in this exudate by using the Grocott methenamine silver staining technique and IHC for *B*. *dermatitidis*. Polyclonal antibody (Meridian Diagnostics, Cincinnati, OH, USA) used in this assay is broadly reactive with multiple fungal species, including *B*. *dermatitidis*, making it useful for detection, but not speciation, of yeasts in tissues.

**Figure 2:**
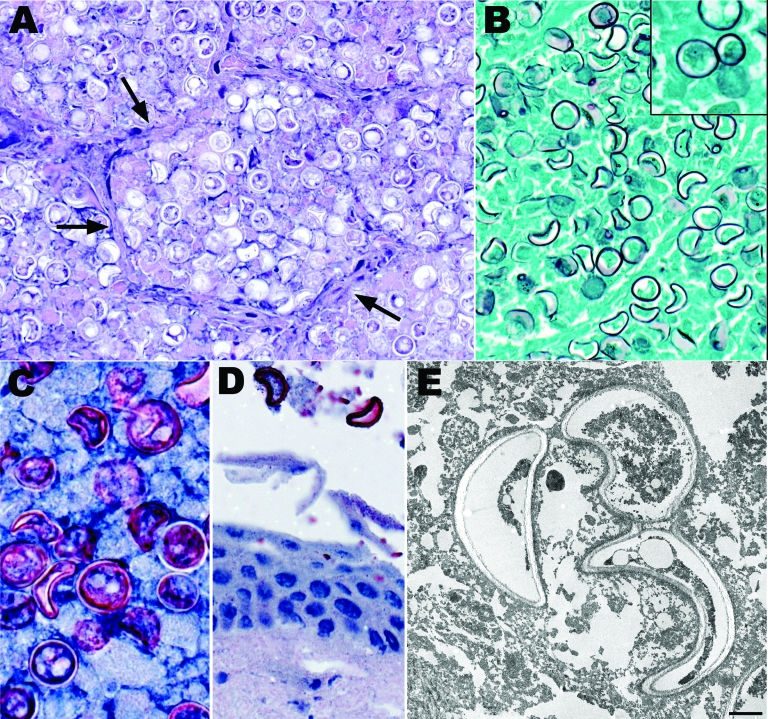
Histopathologic and electron microscopic appearance of *Blastomyces dermatitidis* in kinkajou (*Potos flavus*) tissues. A) Lung showing *B*. *dermatitidis* yeast forms filling alveolar spaces. Alveolar septa are indicated by arrows. B) Lung showing yeast forms of *B*. *dermatitidis*. Inset shows broad-based budding of a yeast form, a major diagnostic feature. C) Lung showing *B*. *dermatitidis* yeast. D) Oral mucosa showing 2 yeast forms of *B*. *dermatitidis* closely associated with the mucosal surface. E) Transmission electron micrograph showing 3 yeast forms of *B*. *dermatitidis* in lung tissue. Note the thick cell walls and crescent shapes of the yeast (scale bar = 2 µm). Hematoxylin and eosin stain (A), Grocott methenamine silver stain (B and inset), and immunoalkaline phosphatase with antibody against *B*. *dermatitidis* and naphthol fast-red with hematoxylin counterstain (C, D). Original magnifications ×400 (A, B, D) and ×630 (Inset, C).

Kinkajou lung tissue was processed for culture and histopathologic and molecular analysis. Staining of lung with hematoxylin and eosin (Figure 2) showed numerous intraalveolar yeasts with double-contoured walls diffusely filling alveolar spaces and associated with inflammatory cell infiltrates (macrophages, lymphocytes, and plasma cells). Yeasts were also closely associated with surfaces of the tongue, palate, and buccal mucosae. Use of the Gomori methenamine silver staining technique and IHC for *B*. *dermatitidis* showed many yeasts in the lungs and fewer in the liver and on epithelial surfaces of the oral cavity.

DNA was amplified from patient tissue and kinkajou lung by using non-nested PCR and primers BlastoI and BlastoII as described (*14*). Pairwise sequence alignment showed that the *B*. *dermatitidis* BAD-1 promoter region sequences from the patient isolate and the kinkajou tissue were indistinguishable.

## Conclusions

The successive timing of the kinkajou’s illness and the patient’s symptom onset suggests that the source of the patient’s infection was the kinkajou bite. Because asymptomatic animal infections are not known to occur (*15*), we believe that the kinkajou likely acquired the infection while living with the patient.

Immediate necropsy and histopathologic analysis should be conducted for any animal that bites a human and then dies, particularly when a lesion develops at the bite site. This report emphasizes the need for early and complete animal necropsy and consideration of nonbacterial etiologies when treating a patient with a recalcitrant infection and a history of an animal bite.
